# Predictors of recovery from severe acute malnutrition among 6–59 months children admitted to a hospital

**DOI:** 10.3389/fpubh.2024.1258647

**Published:** 2024-04-19

**Authors:** Assefa Andargie, Segenet Zewdie

**Affiliations:** ^1^Division of Epidemiology and Biostatistics, Department of Public Health, Injibara University, Injibara, Ethiopia; ^2^Division of Social Pharmacy, Department of Pharmacy, Injibara University, Injibara, Ethiopia

**Keywords:** predictors, recovery, severe acute malnutrition, children, Ethiopia

## Abstract

**Background and aim:**

Severe acute malnutrition is a threat to child survival as mortality rates in children with severe malnutrition are nine times higher. Globally, about 19 million children are severely malnourished. This study looked at children aged 6–59 months admitted to hospital to see how quickly they recovered from severe acute malnutrition as well as what factors predicted their recovery.

**Methods:**

The study included 543 systematically chosen children with severe acute malnutrition who were admitted to the stabilization center of a hospital. Data from the patient registry were gathered using a retrospective follow-up study design. In order to find predictors of recovery, the Cox proportional hazard model was applied.

**Results:**

From 543 children, 425 (78.27%) were recovered. The median survival time was 8 days. Having grade II edema, grade III edema, and pneumonia were negatively associated with recovery. Similarly, taking ceftriaxone, cloxacillin, and being on a nasogastric tube were associated with poor recovery. Conversely, better recovery rates were linked to exclusive breastfeeding and vitamin A supplementation.

**Conclusion:**

Both the recovery rate and the median survival time fell within acceptable bounds. To boost the recovery rate, efforts are needed to lessen comorbidities.

## Introduction

In underdeveloped nations, malnutrition is a common cause of disease and mortality. Malnutrition is a cause in about 45% of deaths in children under the age of five (1, 2). Severe Acute Malnutrition (SAM), as defined by the World Health Organization (WHO), includes severe wasting and nutritional edema. Severe wasting is defined as weight-for-height (WFH) or weight-for-length (WFL) below −3 standard deviations (SD or 𝑍-scores) of the median of the WHO growth standards or mid-upper arm circumference (MUAC) <115 mm (3, 4).

Globally, there were 45 million (6.8%) wasted children under five in 2022, with 13.7 million (2.1%) of them classified as severely wasted. Because of its widespread occurrence and link to child mortality, SAM is the most significant nutritional problem in underdeveloped nations. Asia was home to more than two thirds (70%) of all wasting children under five, while Africa accounted for more than one-quarter (27%) of the global prevalence. In Africa, about 12.2 million children under five were wasted of which 3.5 million were in East Africa (2).

In Ethiopia, about 7% of children are wasted, and 1% are severely wasted. Wasting, or acute malnutrition is highest in children less than 6 months of age and children aged 12–17 months (9.6 and 8.9%, respectively) and lowest in children aged 36–47 months (4.4%). Male children are slightly more likely to be wasted (8.8%) than female children (5.5%). Wasting is high in children from the Somali region (21%), in children whose mothers have no education (9.2%), and in children from the poorest households (11.7%) (5).

The length of hospital stays (LoS) plays a crucial role in effective hospital management and patient care. Accurate measurement of LoS allows hospitals to allocate resources efficiently. By understanding how long patients stay, hospitals can plan for staffing, bed availability, and other essential resources. LoS directly impacts hospital costs. A shorter stay reduces expenses related to inpatient care, medications, and other services. Conversely, longer stays can strain hospital budgets (6). Monitoring LoS helps assess the quality of care provided. Longer stays may indicate issues such as poor communication, medical errors, or inadequate discharge planning. Hospitals strive to minimize LoS while maintaining high-quality care (6, 7). Hospitals use average LoS as an efficiency indicator. A well-managed hospital aims for shorter stays without compromising patient well-being. Efficient care benefits both patients and healthcare systems. Moreover, Hospitals can compare their LoS with national or international averages. This benchmarking helps identify areas for improvement and promotes best practices (6). Studying recovery time from SAM helps to estimate how long it takes for a child to recover from the illness and what factors can affect recovery. It can also help professionals identify ways to improve recovery times and develop better interventions for the illness (8).

The time to recovery from SAM varies across studies. In Ethiopia, various studies determined the recovery time from SAM and produced values ranging from 11 days to 26 days. The time to recovery was affected by many factors including age, sex, residence, the presence of clinical features like fever, diarrhea, vomiting, presence of comorbidities like malaria, HIV, pneumonia, and anemia. The medications and therapeutic feedings used during the management of SAM were also greatly affecting the recovery time (9–21).

Despite the presence of few studies, the recovery time, rate, and predictors of recovery are not consistent across the different studies. The authors hypothesized that the results would differ from those of other studies because the study included cases during the COVID-19 pandemic. Furthermore, instabilities in different parts of the country resulted in a huge internal displacement in the study area, which could have a substantial impact on patient flow, length of stay, and the healthcare delivery system. Therefore, this study was aimed to determine the recovery time, rate and its predictors from SAM among 6–59 months children admitted to Dessie Comprehensive Specialized Hospital located in northeast Ethiopia.

## Materials and methods

### Study design, area, and period

A retrospective follow-up study design was used to determine the time, rate, and predictors of recovery from SAM among 6 to 59 months children admitted to Dessie Comprehensive Specialized Hospital. The hospital is the biggest of all others in northeast Ethiopia which is serving as a teaching and referral center for more than 8 million population in the region. Northeast Ethiopia is known for its recurrent drought and enduring food insecurity. The study included registries from September 2019 to August 2021. The data were extracted from September 12, 2021, to September 30, 2021.

### Population and eligibility criteria

The study population was 6–59 months children with SAM who were admitted to Dessie Comprehensive Specialized Hospital from September 2019 to August 2021. All charts of 6–59 months children with SAM who were admitted to the Hospital from September 2019 to August 2021 were eligible for inclusion. Whereas, incomplete and inaccessible patient registers were excluded.

### Sample size and sampling procedures

The sample size was calculated in Stata 14.1 statistical software using the power and sample size analysis function for the Cox model. Parameters were taken from a previous study conducted in South Wollo (14). From this study, the presence of comorbidities was taken as a factor affecting recovery from SAM. The computation was based on the assumption of a 0.05 level of significance, 80% power, 1.44 hazard ratio, 0.33 standard deviation, and a two-sided test. Thus, the computed sample size was 543. The number of records of children admitted to the hospital from September 2019 to August 2021 was enumerated and samples were selected using a systematic sampling technique.

### Operational definitions

Time-to-recovery: was determined by calculating the difference (in days) from the start of treatment until the child is discharged due to recovery. Cured: children who have reached the discharge criteria for SAM treatment. If admitted with bilateral pitting edema, a child is discharged as cured when there is no bilateral pitting edema for 2 consecutive visits AND MUAC ≥12.5 cm or WFH/WFL ≥ −2 z-scores AND clinically well and alert. If admitted based on WFH/WFL, the child is discharged as cured when: WFH/WFL ≥ −2 z-scores AND no bilateral pitting edema AND clinically well and alert (22). Died: if the child dies while receiving treatment in the stabilization center. Defaulted: a confirmed absence for two consecutive days. Non-responder: does not reach the SAM discharge criteria after 16 weeks of treatment. Stabilized: the condition has stabilized and referred to continue treatment in OTP. Transferred-out: moved to another facility for further medical care. Recovered: children who were cured and stabilized together. Censored: children whose treatment outcome is other than recovered.

### Data collection procedures

The data were extracted from patient charts using a semi-structured extraction form by four trained data collectors. The data extraction format included the child’s characteristics on admission, follow-up information, and the treatment outcome on the date of discharge. Variables like age, sex, residence, and admission type were recorded. The history and physical examination findings including anthropometric measurements (height/length, weight, MUAC), presence of other comorbidities, routine medication administration, and therapeutic feeding provisions were cautiously extracted. The treatment outcome, weight, and other follow-up interventions were recorded on the date of discharge. The data quality was maintained through the quality monitoring team by checking the filled extraction formats for consistency and completeness of the information. Meanwhile, incomplete and inconsistent extraction formats were rejected.

### Data processing and analysis

After the data extraction process, the data were re-checked for completeness and consistency. The data was entered into a pre-designed format in EpiData entry client version 4.6.0.0. Then, it was transferred to STATA 16 (StataCorp, USA) for analysis. The data underwent a preliminary check to prepare it for further analysis. For the categorical variables, frequencies and percentages were used, and summary statistics were used for the quantitative variables to describe the key findings. The Kaplan-Meir estimator was used in the descriptive survival analysis, and the Log Rank test was used to compare survival curves between the groups. To identify predictors of recovery, the proportional hazard Cox regression model was fitted. Bivariable Cox regression was first carried out at a significance level of 0.20 to identify variables eligible for multi-variable Cox-regression. In the multiple Cox regression, statistical significance was declared at 0.05 *p*-values. The Cox proportional hazard (PH) assumption was examined based on the Schoenfeld residuals (global test) and was insignificant (*p* = 0.7630). The overall model fitness was assessed using the Cox-Snell residual plot. There was no risk of multicollinearity as assessed by variance inflation factor (VIF). The adjusted hazard ratio (AHR) with its 95% confidence interval (CI) was reported to identify the major predictors that affect recovery.

### Ethics approval and consent to participate

Since the data for this study came from patient records and there was no direct interaction with the study participants, ethical approval was not thought. However, to get authorization to access patient records, a letter of cooperation was sent to the hospital. The data were anonymously linked to a code number to maintain confidentiality and hence investigators had no access to information that could identify individual participants during or after data collection. We made sure that all procedures were carried out by the WHO recommendations and the standard operating procedures of the healthcare facility.

## Results

### Characteristics of participants

A total of 543 charts of SAM-affected children aged 6–59 months were included. Of them, 423 (77.9%) lived in a rural area, 322 (59.3%) were male, 227 (41.8%) were between the ages of 12 and 23 months, with a median age of 16.0 (IQR = 15) months, and 227 (41.8%) had been exclusively breastfed for the first 6 months. Regarding their clinical characteristics, no edema was seen in 349 (64.3%) patients, while grade III edema was present in 127 (23.4%), 103 (19.0%) had fever, 190 (35.0%) had diarrhea, 230 (42.4%) had vomiting, and 225 (41.4%) had cough. In addition, 202 (37.2%) children had pneumonia, 25 (4.6%) developed dermatitis, and 236 (43.5%) children were anemic (Table 1).

**Table 1:** Baseline characteristics of children admitted to the nutritional stabilization center of a hospital in Northeast Ethiopia, 2019–2021 (*n* = 543).

Baseline characteristics		Count (%)
Residence	Rural	423 (77.9)
	Urban	120 (22.1)
Sex	Male	322 (59.3)
	Female	221 (40.7)
Age in months	6–11	170 (31.3)
	12–23	227 (41.8)
	24–35	84 (15.5)
	36–47	36 (6.6)
	48–59	26 (4.8)
Level of edema on admission	No edema (0)	349 (64.3)
	Grade I (+)	18 (3.3)
	Grade II (++)	49 (9.0)
	Grade III (+++)	127 (23.4)
Exclusive breastfeeding	Yes	227 (41.8)
	No	316 (58.2)
Fever	Yes	103 (19.0)
	No	440 (81.0)
Diarrhea	Yes	190 (35.0)
	No	353 (65.0)
Vomiting	Yes	230 (42.4)
	No	313 (57.6)
Cough	Yes	225 (41.4)
	No	318 (58.6)
Dehydration	Yes	80 (14.7)
	No	463 (85.3)
Color of conjunctiva	Pale	73 (13.3)
	Pink	471 (86.7)
Anemia	Yes	236 (43.5)
	No	307 (56.5)
Pneumonia	Yes	202 (37.2)
	No	341 (62.8)
Dermatitis	Yes	25 (4.6)
	No	518 (95.4)

### Routine medications

As a routine medication after admission, about 82 (15.1%) received amoxicillin, 414 (76.2%) received ampicillin, 447 (82.3%) received gentamycin, 32 (5.9%) received metronidazole, 184 (33.9%) took ceftriaxone, 50 (9.2%) took ceftazidime, and 75 (13.8%) took Lasix. In terms of nutritional supplements 463 (85.3%) of the patients received F-75, 78 (14.4%) received F-100, 118 (21.7%) took ReSoMal, 97 (17.9%) received folic acid, 90 (16.6%) received zinc oxide, 25 (4.6%) received vitamin A and 81 (14.9%) received vitamin D supplementation, 24 (4.4%) received IV fluids, 120 (22.1%) received oxygen, 207 (38.1%) were on nasogastric tubes, and 57 (10.5%) were given a blood transfusion (Table 2).

**Table 2:** Medications, therapeutic feedings, and life support interventions given to children admitted to the nutritional stabilization center of a hospital in Northeast Ethiopia, 2019–2021 (*n* = 543).

Medications/interventions	Count (%)
Amoxicillin	82 (15.1)
Ampicillin	414 (76.2)
Gentamycin	447 (82.3)
Metronidazole	32 (5.9)
Vancomycin	54 (9.9)
Ceftriaxone	184 (33.9)
Ceftazidime	50 (9.2)
Cloxacillin	31 (5.7)
Lasix	75 (13.8)
F_75	463 (85.3)
F_100	78 (14.4)
ReSoMal	118 (21.7)
Folic acid	97 (17.9)
Zinc oxide	90 (16.6)
Vitamin A	25 (4.6)
Vitamin D	81 (14.9)
Calvitalis	48 (8.8)
Anti-pains	52 (9.6)
IV fluid	24 (4.4)
Oxygen	120 (22.1)
Naso-gastric tube	207 (38.1)
Blood transfusion	57 (10.5)

### Treatment outcomes of SAM

Of the 543 SAM-affected children admitted, 354 (65.2%) were fully cured, 71 (13.1%) stabilized, 70 (12. 9%) stopped taking their medications (defaulted), and 27 (5.0%) died. Overall, 425 (78.3%) children recovered from SAM (Table 3).

**Table 3:** Treatment outcomes of children with severe acute malnutrition admitted to the stabilization center of a hospital in Northeast Ethiopia, 2019–2021 (*n* = 543).

		Count	Percent (95% CI)
Treatment outcome	Cured	354	65.2 (61.1, 69.1)
	Died	27	5.0 (3.4, 7.2)
	Defaulted	70	12. 9 (10.3, 16.0)
	Stabilized	71	13.1 (10.5, 16.2)
	Transferred Out	15	2.8 (1. 7, 4.5)
	Not documented	6	1.1 (0.5, 2.4)
Status	Recovered	425	78.3 (74.6, 81.6)
	Censored	118	21.7 (18. 5, 25.4)

### Incidence rate of recovery from SAM

The total person-time at risk followed was 5,133 person days. The overall rate of recovery was 8.28 (95% CI 7.53, 9.12) per 100 person days. Recovery rate varied across the categories of predictors where 13.99 recoveries per 100 person days being the largest rate among children receiving vitamin A supplementation while the smallest rate was recorded among children treated with vancomycin and ceftazidime (3.19 recovery per 100 person days) (Table 4).

**Table 4:** Recovery rate, median survival time, and the log-rank test of equality of survival functions across categories of variables among children with severe acute malnutrition admitted to a hospital in Northeast Ethiopia, 2019–2021 (*n* = 543).

Variables		Recovery rate per 100 person days (95% CI)	Median recovery time (95% CI)	Log-rank test	
				chi-square	*p*-value
Diarrhea	Yes	9.56 (8.18, 11.18)	8.00 (7.54, 8.46)	5.01	0.025
	No	7.67 (6.80, 8.65)	8.00 (7.34, 8.66)		
Vomiting	Yes	9.82 (8.54, 11.29)	8.00 (7.52, 8.49)	11.40	0.001
	No	7.29 (6.41, 8.31)	8.00 (7.22, 8.78)		
Dehydration	Yes	11.00 (8.68, 13.96)	7.00 (5.77, 8.23)	6.79	0.009
	No	7.91 (7.13, 8.77)	8.00 (7.67, 8.33)		
Pneumonia	Yes	6.86 (5.87, 8.00)	8.00 (7.15, 8.85)	9.12	0.003
	No	9.47 (8.39, 10.68)	8.00 (7.62, 8.38)		
Dermatitis	Yes	5.37 (3.29, 8.76)	14.00 (13.09, 14.91)	7.16	0.007
	No	8.46 (7.68, 9.32)	8.00 (7.67, 8.33)		
Amoxicillin	Yes	10.70 (8.59, 13.32)	7.00 (6.39, 7.61)	6.65	0.010
	No	7.87 (7.08, 8.74)	8.00 (7.50, 8.50)		
Metronidazole	Yes	4.23 (2.59, 6.91)	19.00 (10.66, 27.35)	8.78	0.003
	No	8.60 (7.81, 9.48)	8.00 (7.66, 8.33)		
Vancomycin	Yes	3.19 (2.20, 4.62)	19.00 (11.31, 26.69)	46.39	<0.001
	No	9.33 (8.45, 10.29)	8.00 (7.68, 8.32)		
Ceftriaxone	Yes	6.67 (5.63, 7.91)	10.00 (8.34, 11.66)	20.65	<0.001
	No	9.30 (8.29, 10.43)	7.00 (6.64, 7.37)		
Ceftazidime	Yes	3.19 (2.19, 4.65)	19.00 (10.46, 27.54)	43.33	<0.001
	No	9.29 (8.42, 10.25)	8.00 (7.67, 8.32)		
Cloxacillin	Yes	4.88 (3.25, 7.35)	12.00 (10.27, 13.74)	10.19	0.001
	No	8.62 (7.82, 9.51)	8.00 (7.66, 8.34)		
Folic acid	Yes	10.94 (8.75, 13.68)	7.00 (6.35, 7.65)	8.65	0.003
	No	7.86 (7.07, 8.73)	8.00 (7.47, 8.54)		
Vitamin A	Yes	13.99 (9.02, 21.68)	6.00 (4.55, 7.45)	13.37	<0.001
	No	8.12 (7.36, 8.95)	8.00 (7.68, 8.32)		
Vitamin D	Yes	6.08 (4.81, 7.69)	9.00 (7.47, 10.53)	11.13	0.001
	No	8.92 (8.03, 9.89)	8.00 (7.64, 8.36)		
Calvitalis	Yes	6.66 (4.88, 9.08)	11.00 (8.25, 13.75)	7.15	0.007
	No	8.50 (7.69, 9.39)	8.00 (7.66, 8.34)		
Oxygen	Yes	5.87 (4.72, 7.29)	10.00 (8.30, 11.70)	14.16	<0.001
	No	9.17 (8.25, 10.19)	8.00 (7.69, 8.31)		
NG Tube	Yes	7.06 (6.03, 8.27)	9.00 (8.24, 9.77)	19.70	<0.001
	No	9.17 (8.14, 10.33)	7.00 (6.53, 7.47)		
Overall		8.28 (7.53, 9.12)	8.00 (7.67, 8.33)		

### Survival time

**Figure 1:**
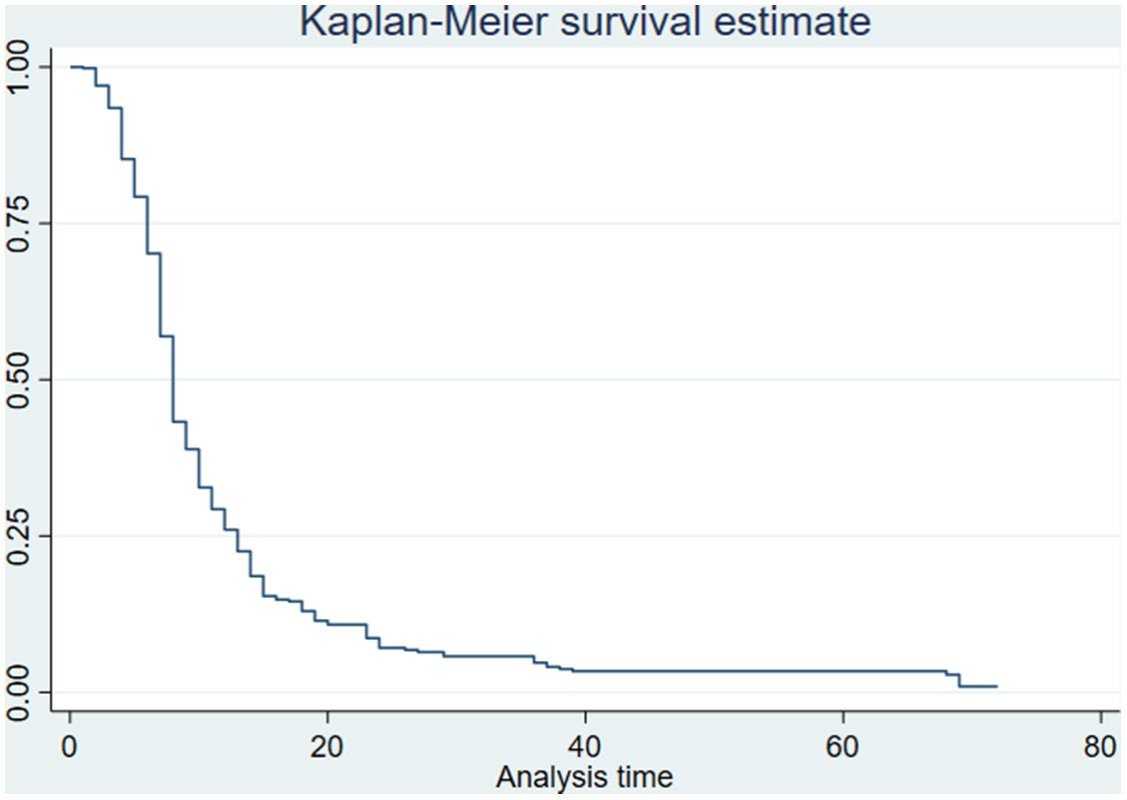
The Kaplan-Mier curve for the survival function of recovery time among children with severe acute malnutrition in Northeast Ethiopia, 2019–2021.

The overall median survival time (time to recovery from SAM) was found to be 8 days (95% CI: 7.67, 8.33). A statistically significant difference in the survival function was observed between groups of predictors as shown in the log-rank test result (Table 4; Figure 1).

### Predictors of recovery from SAM

The bivariate Cox regression showed associations with recovery at the 0.20 level of significance for age, level of edema, the presence of fever, diarrhea, exclusive breastfeeding, dehydration, anemia, pneumonia, usage of vancomycin, ceftriaxone, cloxacillin, folic acid, vitamin A, vitamin D, and an NG tube. Where as in the multiple Cox regression analysis, the presence of pneumonia, the degree of edema, exclusive breastfeeding dehydration, the use of antibiotics such as ceftriaxone and cloxacillin, vitamin A, and an NG tube were all found to be statistically significant predictors of SAM recovery.

Children with grade II edema had a 41% lower chance of recovering than children without edema (AHR = 0.59, 95% CI: 0.40, 0.88). Similarly, children with grade III edema were 31% less likely to recover than children with no edema (AHR = 0.69, 95%CI: 0.51, 0.92). Children who were exclusively breastfed until the age of 6 months had a 34% higher chance of recovering than those who were not (AHR = 1.34, 95%CI:1.05, 1.71). Children who had pneumonia had a 31% lower chance of recovering compared to those who did not (AHR = 0.69, 95%CI: 0.56, 0.86). Ceftriaxone use among children was associated with a 42% lower likelihood of recovery compared to ceftriaxone-free children (AHR = 0.58, 95%CI: 0.45, 0.73). Children who were taking cloxacillin had a 47% lower chance of recovering compared to those who were not (AHR = 0.53, 95%CI: 0.33, 0.86). The rate of recovery for children taking vitamin A was 2.13 times higher than it was for children who were not taking it (AHR = 2.13, 95%CI: 1.28, 3.54). When compared to children without NG tubes, those on NG tubes had a 33% lower chance of recovering (AHR = 0.67, 95%CI: 0.53, 0.84) (Table 5).

**Table 5:** The predictors of recovery among children with severe acute malnutrition admitted to a hospital in Northeast Ethiopia, 2019–2021 (*n* = 543).

Predictors		Number at risk	Recovered (*N*, %)	CHR (95% CI)	AHR (95% CI)	*p*-value
Age (months)	6–11	170	125 (73.5)	1.00	1.00	
	12–23	227	192 (84.6)	1.23 (0.98,1.55)	1.08 (0.83, 1.39)	0.586
	24–35	84	69 (82.1)	0.92 (0.69,1.24)	0.87 (0.63, 1.20)	0.396
	36–47	36	27 (75.0)	0.81 (0.53,1.24)	0.64 (0.39, 1.05)	0.075
	48–59	26	12 (46.2)	1.04 (0.58,1.89)	1.33 (0.69, 2.58)	0.397
Level of edema	No edema	349	279 (79.9)	1.00	1.00	
	Grade I	18	13 (72.2)	1.09 (0.62,1.90)	0.68 (0.34, 1.35)	0.268
	Grade II	49	31 (63.3)	0.74 (0.51,1.08)	0.59 (0.40, 0.88)	0.010*
	Grade III	127	102 (80.3)	0.93 (0.74,1.16)	0.69 (0.51, 0.92)	0.011*
Exclusive breastfeeding	Yes	227	192 (84.6)	1.17 (0.97,1.42)	1.34 (1.05, 1.71)	0.017*
	No	316	233 (73.7)	1.00	1.00	
Presence of fever	Yes	103	77 (74.8)	0.81 (0.63,1.04)	0.89 (0.65, 1.22)	0.477
	No	440	348 (79.1)	1.00	1.00	
Diarrhea	Yes	190	158 (83.2)	1.23 (1.01,1.51)	1.21 (0.95, 1.53)	0.116
	No	353	267 (75.6)	1.00	1.00	
Dehydration	Yes	80	68 (85.0)	1.38 (1.06,1.79)	1.14 (0.82, 1.58)	0.434
	No	463	357 (77.1)	1.00	1.00	
Anemia	Yes	236	194 (82.2)	1.14 (0.94,1.38)	1.07 (0.86, 1.34)	0.540
	No	307	231 (75.2)	1.00	1.00	
Pneumonia	Yes	202	160 (79.2)	0.75 (0.61,0.92)	0.69 (0.56, 0.86)	<0.001*
	No	341	265 (77.7)	1.00	1.00	
Vancomycin	Yes	54	28 (51.9)	0.30 (0.20,0.45)	0.70 (0.24, 2.02)	0.508
	No	489	397 (81.2)	1.00	1.00	
Ceftriaxone	Yes	184	133 (72.3)	0.64 (0.52,0.79)	0.58 (0.45, 0.73)	0.001*
	No	359	292 (81.3)	1.00	1.00	
Cloxacillin	Yes	31	23 (74.2)	0.53 (0.35,0.81)	0.53 (0.33, 0.86)	0.005*
	No	512	402 (78.5)	1.00	1.00	
Folic acid	Yes	97	77 (79.4)	1.42 (1.10,1.82)	1.27 (0.93, 1.72)	0.128
	No	446	348 (78.0)	1.00	1.00	
Vitamin A	Yes	25	20 (80.0)	2.17 (1.38,3.42)	2.13 (1.28, 3.54)	0.003*
	No	518	405 (78.2)	1.00	1.00	
NG Tube	Yes	207	153 (73.9)	0.66 (0.54,0.80)	0.67 (0.53, 0.84)	<0.001*
	No	336	272 (81.0)	1.00	1.00	

*Statistically significant at α = 0.05. CHR, Crude hazard ratio; AHR, Adjusted hazard ratio; CI, Confidence interval.

## Discussion

In developing nations, severe acute malnutrition is one of the most frequent reasons for hospitalization in children. The purpose of this study was to determine the length, rate, and predictors of SAM recovery in children between the ages of 6 and 59 months.

In this study, the median time to recovery was 8 days (95% CI: 7.67, 8.33). The result is consistent with studies from Waghimra (21), East Amhara (13), Gondar (23), South Wollo (14), and North Shoa (24) hospitals. However, it is lower than a study conducted in Pawi (14 days) (9), Axum (15 days) (25), Jimma University medical center (19 days) (19), Addis Ababa St. Paul’s Hospital (17 days) (26), Amhara region stabilization centers (16 days) (17), Addis Ababa Yekatit 12 hospital (15 days) (17, 27), Southern Ethiopia (17 days and 26 days) (27, 28), and Bahir Dar Felege Hiwot referral hospital (16 days and 18 days) (18, 29). In a similar vein, the median recovery time falls within the acceptable range of global guidelines established by the SPHERE initiative (28 days) (30). This quick recovery period can be seen as encouraging progress in the research area’s quest for high-quality healthcare.

In this study, the percentage of children who had recovered from SAM was reported to be 78.3% (95% CI: 74.6, 81.5). The result complies with the SPHERE project’s international standard criteria, which is set at >75% recovery (30). It also accords with research done in Ethiopia, notably at Waghimra (21), Eastern Amhara (13), Jimma University medical center (19), Addis Ababa St. Paul’s Hospital (26), Addis Ababa Yekatit 12 Hospital (20), South Wollo (14), and Southern Ethiopia (28). In contrast, it is higher than studies done at the University of Gondar Hospital (23) Bahir Dar Felege Hiwot Hospital (29), North Shoa (24), Southern Ethiopia (27), Axum (25), Pawi hospital (9), and Dubti hospital (12). Unlike some of the other studies listed, this study’s definition of success included both cured and stabilized, as opposed to some of the others that only included a complete cure.

Various factors affected the recovery rate differently. Compared to children without edema, children with grade II and grade III edema had a worse chance of recovering. This finding is similarly supported by results from comparable studies (17, 29). The rationale that might be used perhaps the children with edema have additional issues like chronic cardiac conditions that take longer to recover from.

Children who had pneumonia were less likely to recover from SAM compared to those who had no pneumonia. This finding was supported by the outcomes of other studies. In comparison to cases where there are no comorbidities, those with comorbidities like pneumonia may require longer and more cautious follow-up care. Additionally, children with comorbidities are more likely to be referred to advanced care facilities for additional treatment, which results in an underestimation of the study area’s recovery rate. Based on this finding, healthcare providers should develop a new SAM management strategy for children with pneumonia in order to speed up their recovery.

One of the important factors that increased the likelihood of recovery was exclusive breastfeeding. Children who exclusively breastfed until 6 months had a 34% higher rate of recovery than their counterparts. Studies carried out at Pawi Hospital and Addis Abeba St. Paul’s Hospital provide evidence in support of this conclusion. Evidence suggests that up to 6 months of exclusive breastfeeding can shield a kid from respiratory infections and diarrheal illnesses. The presence of comorbidities like pneumonia reduces the rate of recovery as evidenced by findings from this study. Therefore, promoting exclusive breastfeeding for the first 6 months is important to improve recovery from SAM.

Routine medications including ceftriaxone and cloxacillin are associated with delayed recovery rate. All children admitted with severe malnutrition are presumed to be infected, and broad-spectrum antibiotics are advised for their treatment. These medications are in general given to children who had further complications, infections, and/or other comorbidities which may attribute to longer recovery time (3, 31, 32). Improving recovery may require lowering the risk of complications and other comorbidities.

The recovery rate among children supplemented with vitamin A was shorter than their counterparts. This finding is supported by studies conducted in Addis Ababa (26), Amhara region (17), and Bahir Dar (29). All severely malnourished children have Vitamin A deficiency (31, 32). Vitamin A supplementation for severely malnourished children is a multi-purpose strategy. It may be given to prevent its deficiency, treat an identified deficiency, prevent infection, improve immunity, and facilitate tissue regeneration (33).

Children who were on NG tube were less likely to recover compared to children without NG tube. The finding is similar to a study conducted in East Amhara hospitals. NG tube is indicated for children who had a loss of appetite, complications, and other life-threatening conditions that halt feeding per oz. these conditions in turn need further management procedures and follow up leading to longer recovery time.

The key takeaways from the study’s findings are on how crucial it is to avoid comorbidities and consequences. The fact that the children have pneumonia, are on medication, and are on NGT suggests that they were admitted with a complicated SAM or comorbidities. To deal with preventing complications and comorbidities is crucial for professionals and parents as all these disorders reduce the recovery rate.

This study has the limitation of being a retrospective analysis of patient records. This approach would not offer all the data required for research purposes. Parental and other sociocultural factors that can affect the time to recovery were also overlooked. Even so, it can still offer crucial evidence to pinpoint predictors linked to the child and the SAM management system. Additionally, because the study is restricted to a single institution, care must be used when drawing inferences. Despite these limitations, the finding is nevertheless crucial for directing local policies and actions.

## Conclusion

The rate of recovery for children with SAM was found to be 78.3% which is within the international recommended percentage of recovery (>75%). Compared to other studies, the median recovery time of 8 days was optimal. The likelihood of recovering from SAM was significantly predicted by the existence of edema, exclusive breastfeeding, pneumonia, the use of ceftriaxone, cloxacillin, and vitamin A, as well as the presence of an NG tube. Healthcare institutions and professionals should pay special attention while treating SAM in children with pneumonia and advanced complications to improve the recovery rate. The number of hospitalizations owing to complicated SAM may be decreased by raising community awareness of the early indications of child malnutrition, as early detection speeds up recovery. Strengthening vitamin A supplementation in both the community and institutions is important since it speeds up recovery. It is recommended that researchers carry out prospective follow-up studies with familial backgrounds included to ascertain their impact on the recovery rate of children from SAM.

## Data availability statement

The data analyzed in this study is subject to the following licenses/restrictions: The data can be accessed from the author upon request. Requests to access these datasets should be directed to assefaand@gmail.com.

## Author contributions

AA: Data curation, Formal analysis, Investigation, Methodology, Writing – original draft. SZ: Conceptualization, Supervision, Validation, Writing – review & editing.
